# DSM-5: problems and suggestions

**DOI:** 10.1186/2050-2974-1-8

**Published:** 2013-02-25

**Authors:** Andreas Birgegård, Gaby Groß, Joakim de Man Lapidoth, Claes Norring

**Affiliations:** 1 Department of Clinical Neuroscience, Karolinska institute, 171 76, Stockholm, Sweden; 2 Department of Psychosomatic Medicine and Psychotherapy, University Hospital Tübingen, Osianderstr. 5, 72076, Tübingen, Germany

**Keywords:** Diagnostics, Anorexia, Bulimia

## Abstract

The upcoming DSM5 will impact clinical work and research substantially, and we here point to a few problems that may have been overlooked. These concern inconsistencies and lack of clarity, and the future “not elsewhere classified” atypical anorexia nervosa category. We propose solutions in the form of working definitions and operationalizations to facilitate clinical implementation as well as streamline research.

## 

Dear Editor

Although research into the effects of the DSM-5 expands rapidly, we wish to point out a few issues that are not without consequence but that may have been overlooked. We are aware of the monumental task that the DSM-5 eating disorders (ED) Work Group has faced, with the near irreconcilable concerns involved, but let’s be frank: The ED section in DSM-5 is probably not to everyone’s satisfaction. However, no major changes to the criteria will occur from now on: Production is in its final stages and the proposal on http://www.dsm5.org (recently removed from public access unfortunately) is probably definite. We may be accused of over-detailed scrutiny, especially by clinicians who daily have to reconcile the letter of manualized criteria with fluid and unwieldy reality. However, there is one instance – that will affect clinical work and research alike – which requires the kind of nitpicking we have possibly perpetrated, namely the adaptation of diagnostic instruments to DSM-5.

In the process of just such an undertaking, we have noted the following:

1. An inconsistency between the second part of the C criterion for anorexia nervosa (AN) and the D criterion for bulimia nervosa (BN). The former reads “Undue influence of body weight *or* shape on self-evaluation”, while the latter reads “Self-evaluation is unduly influenced by body shape *and* weight” (emphasis added). We would suggest that the AN version be used for both, i.e. either the one or the other, to be equally inclusive for both disorders.

2. Does AN “restriction of energy intake” concern both food intake and uptake (since energy, not food, is mentioned)? For example, can it include purging, or diet pills, thereby preventing calorie uptake? We suggest that uptake should be intended, and that purging or other ways of preventing calorie absorption can fulfill the criterion, even though food is consumed.

3. Enemas are mentioned as a purging example in AN, but not in BN, and “mere” examples, as so often happens with manuals, may be reified and lead to assessment differences. We suggest that enemas, although rather rarely used, are relevant for BN also.

4. Significant weight loss, as well as its time frame and velocity, are not defined in the “not elsewhere classified” Atypical AN (AAN), possibly in keeping with the Work Group reluctance to define numeric limits, as in AN weight. This can cause problems for clinical use and research. For example, can it include both a 10% weight loss over the last 3 months and a 20% loss that stabilized 9 months ago? Agreeing on a working definition would be of great value in research that wants to use the category but not specifically investigate the weight loss issue, and for clinicians who need to decide whether an ED is present.

5. Continuing on AAN, the B and C criteria for AN proper (both required for AAN) need clarifications:

a. The B criterion states that fear of weight gain or becoming fat, or persistent behavior that interferes with weight gain should be present, *even though at a significantly low weight*. If this criterion applies *only* to low weight patients, it does not apply to AAN. Since fear/avoidance of weight gain is common also in patients with restricting eating pathology, but without significantly low weight, the criterion should be applicable also for AAN.

b. In the three-part C criterion, the last part (“persistent lack of recognition of the seriousness of the current low body weight”) obviously also hinges on a low weight and is thus irrelevant for AAN. The other two parts however (disturbance of body shape/weight experience and undue influence of shape/weight on self-evaluation), work as general ED symptoms and should be retained for AAN as an either/or criterion, although a reliable operationalization of body experience disturbance for non-underweight people may prove elusive, and this issue deserves research attention.

As a consequence, the AAN description may be reworded into: *Despite significant weight loss, the individual’s weight is within or above the normal range, and there is a) an intense fear of gaining weight or becoming fat, or persistent behavior that interferes with weight gain, and b) a disturbance in the way in which one's body weight or shape is experienced, or undue influence of body weight or shape on self-evaluation*. Whether such a definition truly qualifies as an ED is an empirical question, but the lack of clarity may cloud the issue. We offer our reworded definition to help streamline operationalizations.

As noted, this level of detail may not matter greatly in everyday clinical decisions, but formally, all use of the criteria are affected, or at least we should expect them to be, when constructing the diagnoses. We hope our comments may possibly be relevant for the final DSM-5 texts in some instance, but if not they may be helpful for clinical and research colleagues. The more we use the DSM alike, the better off our attempts at cumulative research will be.

## Competing interests

The authors have no competing interests regarding the present contribution.

## Authors’ contribution

All authors contributed to the arguments made in the manuscript and collaborated on writing, and all authors read and approved the final manuscript.

